# Frailty in Older Adults with Cardiovascular Disease: Cause, Effect or Both?

**DOI:** 10.14336/AD.2017.1125

**Published:** 2018-06-01

**Authors:** Emma EF. Kleipool, Emiel O. Hoogendijk, Marijke C. Trappenburg, M. Louis Handoko, Martijn Huisman, Mike JL. Peters, Majon Muller

**Affiliations:** ^1^Department of Internal medicine and Geriatrics, VU University Medical Center, 1081 HV Amsterdam, The Netherlands; ^2^Department of Epidemiology and Biostatistics, Amsterdam Public Health research institute, VU University Medical Center, 1007 MB Amsterdam, The Netherlands; ^3^Department of Cardiology, VU University Medical Center, 1081 HV Amsterdam, The Netherlands; ^4^Department of Sociology, VU University, 1081 HV Amsterdam, The Netherlands

**Keywords:** Frailty, older adults, cardiovascular disease, heart failure

## Abstract

Cardiovascular disease (CVD) has been associated with an increased risk of frailty, but the direction of the association remains unclear. This study set out to examine the bidirectional longitudinal association between CVD and frailty over an extended period of time. Data are from 1432 older adults (aged 65-88yrs) of the Longitudinal Aging Study Amsterdam (LASA), who were followed for 17 years. At baseline and follow-up, CVD was assessed through self-report, medication use and medical records, and classified as angina pectoris, myocardial infarction, heart failure (HF), stroke, and peripheral artery disease. Throughout the study, frailty was assessed using Fried’s frailty criteria. Cox regression models showed that patients with HF had an increased frailty risk (HR 2.7; 95%CI: 1.5-5.1) after a median follow-up of 8.4 yrs. This finding was independent of potential confounders (age, sex, several comorbidities). Examinations of the reverse association revealed that frail older adults were not at risk of incident CVD. Of all older adults with CVD, those with HF have an increased risk of frailty and frail older adults do not have an increased risk of CVD. Our findings emphasize the need for cardiac rehabilitation programs evaluating the effect of physical exercise programs in order to prevent frailty and therewith improve quality of life and independence of care in CVD patients.

In community-dwelling older adults both cardiovascular disease (CVD) and frailty are highly prevalent. Novel and advanced cardiovascular therapeutic treatments have improved life expectancy and consequently led to an increasing number of older adults suffering from chronic CVD [1]. This presents an enormous clinical and public health burden for Western society [2]. Frailty describes a state of vulnerability due to an age-related decline in many physiological systems [3-5] and is associated with a considerably increased risk of falling, disability, hospitalisation and mortality [6, 7]. According to cross-sectional data, CVD appears to be positively associated with frailty in community-dwelling older adults [8-10]. However, cross-sectional studies do not clarify if CVD leads to frailty or if frailty precedes the development of CVD. From a pathophysiological point of view, both directions are plausible. For example, exercise related symptoms in patients with CVD (e.g. claudication intermittent or exercise induced chest pain) could lead to physical inactivity making them more likely to become frail. Additionally, comorbidities, as well as physical and cognitive decline are common in older adults with CVD. This could lead to a loss of homeostatic capability to withstand stressors and increase the risk of frailty. Yet, one could also argue that physical inactivity and its sequelae (e.g. obesity) due to frailty is a risk factor for development of CVD. Also, frailty is associated with a chronic state of low-grade inflammation [11] which could trigger CVD. Thus, the question if CVD precedes frailty or if frail older adults have an increased risk of CVD remains to be answered. Therefore, we set out to investigate the bidirectional longitudinal relation between CVD and frailty in community-dwelling older adults who were followed for 17 years.

## MATERIALS AND METHODS

### Study subjects

Data from the Longitudinal Aging Study Amsterdam (LASA) were used. LASA is an ongoing multidisciplinary cohort study on emotional, physical, cognitive and social functioning in the older Dutch population. Men and women (aged 55-85 years), stratified by age, sex, urbanization grade and expected 5-year mortality rate were randomly selected from eleven municipal registries in three different regions in the Netherlands. At baseline in 1992/93, 3107 persons were enrolled. Follow-up measurements were collected every three years. Data are collected in a face-to-face main interview and in a separate medical interview (including clinical tests) at the respondent’s home by trained interviewers. Details on the sampling and data collection have been described elsewhere [12, 13]. Individuals born in or before 1930 (aged 65 years and older as of January 1,1996), who participated in the second interview in 1995/96, with information on CVD and frailty, were selected for the current study (n=1432). Since various instruments that are used to assess frailty were included after the first LASA measurement wave (1992/3) and because CVD was checked using the medical files of the subject’s general practitioner in 1995/96, the second wave (1995/6) provided more data than the first wave and was therefore used as baseline measurement. After this second wave (Timepoint 1, T1=1995/96), subjects were followed over a period of 17 years during which follow-up measurements were collected approximately every three years. For the present study, we used data of 5 follow-up cycles (T2=1998/9, T3=2001/2, T4=2005/6, T52008/9, T6=2011/2). The Medical Ethics Committee of the VU University Medical Center approved the study and all persons gave written informed consent. The study was conducted in accordance with the principles of the Declaration of Helsinki.

### Frailty

Measurements and cut-offs identical or similar to Fried’s frailty criteria [3] were used to assess frailty at baseline and during every follow-up measurement (T1-T6). This phenotype includes weight loss, weak grip strength, exhaustion, slow gait speed and low physical activity. Subjects were considered not frail if less than three of the five criteria were present and frail if three or more criteria were present. We applied Fried’s original cut-off scores for the individual phenotype components, except for gait speed and physical activity in which we used the lowest quintile approach [14]. This slightly modified frailty phenotype has been validated for mortality in the LASA study [15]. Weight loss was present if a subject lost 5% or more body weight in the previous three years [16]. Body weight was measured without clothes and shoes using a calibrated bathroom balance scale to the nearest 0,1 kg. Height was measured using a stadiometer, to the nearest mm. Body mass index (BMI) was calculated by taking measured body weight in kilograms and dividing it by measured height in meters squared. Grip strength was assessed with a handheld dynamometer (Takei TKK5001; Takei Scientific Instruments, Tokyo, Japan). The sum of the highest values of two measurements on each hand was used. Original cut-off points stratified by sex and BMI were applied to indicate weak grip strength [3]. Exhaustion was measured using two questions from the Center for Epidemiologic Studies Depression Scale (CES-D) [17]. The exhaustion criterion was considered present if a subject answered “often” or “most of the time” to the following two statements: “In the last week I felt that everything I did was an effort” and “In the last week I could not get going.” Gait speed was assessed by recording the time taken (in seconds) to walk 3 meters, turn around, and walk the 3 meters back as quickly as possible [18]. Slow gait speed was defined by the lowest quintile, stratified by sex and height. Finally, physical activity was assessed using the LASA Physical Activity Questionnaire (LAPAQ) [19]. Low physical activity was defined by the lowest quintile of average time spent on physical activities (walking and cycling) per day during two weeks before the interview. Cut-offs derived from data at baseline were used across all follow-up measurements [15].

### Cardiovascular diseases

Presence of angina pectoris (AP), myocardial infarction (MI), heart failure (HF), peripheral arterial disease (PAD), and stroke was assessed at baseline (T1) and during the first follow-up cycle after three years (T2). An algorithm was made for each particular CVD. Presence of these CVDs was proved satisfactory if at least two of the following three criteria were present: (1) self-reported (symptoms of) CVD, (2) use of disease specific medication during the past 2 weeks or (3) medical records of the general practitioner [20]. If one or more of the specific CVDs was present, CVD (combined variable) was scored ‘yes’.

### Cardiovascular risk factors, comorbidities and polypharmacy

The following cardiovascular risk factors were assessed: age, sex, systolic and diastolic blood pressure, nutritional status (based on BMI), use of antihypertensive medication and statins, smoking status, alcohol use, total cholesterol, LDL cholesterol, HDL cholesterol, and triglycerides. Data on sex and age were derived from population registries. Blood pressure was measured in sitting position using a standard mercury sphygmomanometer (Omron HEM706 automatic device). Smoking status (never, former, current smoker) and alcohol use were assessed by standardized questionnaires [21]. Alcohol use was classified by the Garretsen index (does not drink, light, and moderate/excessive drinker) [22]. Blood samples were drawn in the morning, processed, and centrifuged within 60 minutes. Samples were kept frozen at -80°C until determination by the Department of Clinical Chemistry of the VU University medical center. Questions concerning co-morbidity were included in the interview using the chronic disease questionnaire of Statistics Netherlands [21]. Subjects were asked whether they currently or previously had one of the following chronic diseases: chronic lung disease (asthma and chronic obstructive pulmonary disease), arthritis (rheumatoid arthritis or osteoarthritis), cancer, and urine incontinence. Presence of diabetes mellitus was based on an algorithm combining self-report with records of a subject’s general practitioner and diabetes specific medication. Subjects were asked to collect all containers of medication they were currently using (prescribed by a physician and over-the-counter drugs). The interviewer registered names and dosages. The anatomical-therapeutic-chemical (ATC) coding and categorization system for medication was used to classify these drugs [23], including use of antihypertensive medication and lipid lowering drugs. Polypharmacy was defined as chronic use of five or more medications [24]. Mortality status and date of death were retrieved from registers of the municipalities where respondents lived.

### Statistical analysis

To assess the (bidirectional) association between CVD and frailty the following analyses were conducted: (1) cross-sectional association between CVD and frailty; (2) longitudinal association between CVD and incident frailty; (3) longitudinal association between frailty and incident CVD; (4) time-lag analyses of the associations between CVD and incident frailty and frailty and incident CVD.

#### Cross-sectional association between CVD and frailty

Baseline differences in subjects with CVD versus no CVD were assessed. Depending on the distribution of the variable, T-test, Mann-Whitney U test, and Chi-square Test were used to calculate differences in mean, median, and frequencies respectively. Data on serum cholesterol levels were missing on 187(13%) of the subjects in the cross-sectional analyses and 137(11%) in the longitudinal analyses. Blood pressure (systolic and diastolic) was not measured in 31 subjects (2%). Data on alcohol and smoking were not available for three, respectively two subjects (<1%).

Multivariable logistic regressions were performed to evaluate the cross-sectional relationship between CVD (any CVD and specific CVDs) and other factors known to be associated with frailty: chronic lung disease[25, 26], arthritis[25, 27, 28], cancer[29], diabetes[25], urine incontinence[30], and polypharmacy[31]. Odds ratios (ORs) and 95% confidence intervals (CIs) were estimated for the frail compared with the non-frail. Subjects who had information on CVD and frailty were included in the analyses (n=1432). Initial models were adjusted for age and sex. Secondary models were adjusted for age, sex, comorbidities, and polypharmacy.

#### Longitudinal association between CVD and incident frailty

Subjects who were non-frail at baseline and had information on CVD were included in the analyses (n=1222). Baseline differences in non-frail persons with and without CVD were determined. All subjects were followed up for 12 years (T2-T6) for the occurrence of frailty. Time to frailty was calculated as the time between baseline and the interview date at which subjects were considered to be frail.

Cox proportional hazard models with time to frailty in days were used to calculate hazard ratios (HRs) and 95%CIs for incident frailty in subjects with and without CVD (combined variable and individual CVDs) and in subjects with and without the above-mentioned factors (chronic lung disease, arthritis, cancer, diabetes, urine incontinence, and polypharmacy). Non-frail subjects who died during follow-up were censored at their date of death. Persons surviving with no evidence of frailty were censored at the end of their follow-up. First models were adjusted for age and sex. Secondary models were adjusted for age, sex and comorbidities.

**Table 1:** Baseline characteristics in subjects with and without CVD (n=1432).

	CVD		P-value
	Yes n=148	No n=1284	
Demographics			
Age (yrs) ^a^	78.3 ± 5.9	75.4 ± 6.6	0.01
Sex (%female)	47%	52%	0.28

Frailty score			
Weight loss^b^	32 (22%)	218 (18%)	0.14
Weak grip strength^c^	86 (56%)	470 (36%)	0.00
Exhaustion^d^	46 (30%)	196 (15%)	0.00
Slow gait speed^e^	50 (35%)	231 (18%)	0.00
Low physical activity^f^	50 (35%)	253 (20%)	0.00

Cardiovascular disease^g^			
Angina pectoris	83 (56%)		
Myocardial infarction	16 (11%)		
Heart failure	54 (37%)		
Stroke	14 (10%)		
Peripheral artery disease	14 (10%)		

Cardiovascular risk factors			
Nutritional status^g^			0.03
Low weight (BMI^h^ <20)	2 (1%)	54 (4%)	
Normal weight (BMI 20-25)	35 (24%)	396 (31%)	
Overweight (BMI >25)	111 (75%)	834 (65%)	
Systolic blood pressure (mmHg)^a^	151 ± 28	154 ± 26	0.18
Diastolic blood pressure (mmHg)^a^	81 ± 16	83 ± 13	0.03
Serum cholesterol (mmol/L)^a^			
Total cholesterol	5.6 ± 1.0	5.7 ± 1.0	0.50
LDL cholesterol	3.6 ± 0.9	3.7 ± 1.0	0.43
HDL cholesterol	1.2 ± 0.4	1.4 ± 0.4	0.00
Triglycerides	1.7 ± 0.9	1.5 ± 0.7	0.00
Smoking^g^ Never Former Current	50 (34%) 77 (52%) 20 (14%)	459 (36%) 570 (44%) 254 (20%)	0.10
Alcohol use^g^ No Light Moderate/excessive	45 (31%) 71 (48%) 31 (21%)	311 (24%) 641 (50%) 330 (26%)	0.19
Chronic diseases^g^			
Chronic lung disease	36 (24%)	188 (15%)	0.00
Arthritis	57 (39%)	602 (47%)	0.38
Cancer	19 (13%)	156 (12%)	0.81
Diabetes mellitus	20 (14%)	95 (7%)	0.01
Urine incontinence	54 (37%)	332 (26%)	0.01
Medication^g^ No. of drugs taken 0 1 ≥ 2 Antihypertensive drugs Lipid lowering drugs	3 (2%) 5 (3%) 140 (95%) 126 (85%) 14 (10%)	351 (27%) 287 (23%) 646 (50%) 456 (36%) 51 (4%)	0.00 0.00 0.00

a Mean±standard deviation is presented.

b Loss of 5% or more body weight in the previous 3 years.

c Grip strength in the lowest 20% at baseline, adjusted for gender and BMI.

d Positive score on 2 exhaustion items of the Center for Epidemiologic Studies Depression Scale (CES-D) scale.

e Lowest quintile on 3 meters walking test, stratified bij sex and height.

f Lowest quintile of time spent on physical activities (LAPAQ questionnaire).

g Number of subjects (%).

h Body mass index (kg/m^2^).

#### Longitudinal association between frailty and incident CVD

Data collected at baseline (T1) and during the first follow-up cycle (T2) were used in this analysis. Cox proportional hazard models, adjusted for age and sex, were used to calculate hazard ratios and 95%CIs for incident CVD in subjects with and without frailty at baseline. Subjects who had CVD at baseline were excluded from this analysis. Subjects without CVD who died during the three-year follow-up were censored at their date of death. A total of n=1284 were included in these analyses.

#### Time-lag analyses CVD and incident frailty

To minimize the possibility that underlying comorbidity may explain the relation between CVD and frailty, the analyses between CVD and incident frailty were repeated within strata of follow-up years (0-5 and 6-17 years), assuming that longer follow-up is more suggestive of a causal relation between CVD and frailty. Patients who were frail at baseline or at the first follow-up moment were excluded from these analyses. A total of n=1133 were included in these analyses. These models were adjusted for age and sex.

## RESULTS

### Cross-sectional association between CVD and frailty

Baseline characteristics of subjects with CVD (n=148) and without CVD (n=1284) are presented in table 1. The mean age of patients with CVD at baseline was 78.3 years and 47% of these patients were female. Of these subjects, 83 (56%) had AP, 16 (11%) had experienced a MI, 54 (37%) had HF, 14 (10%) PAD, and 14(10%) had suffered a stroke. Of all 148 patients with CVD, 116 had one specific CVD, 31 had two specific CVDs and 1 had three specific CVDs. In terms of cardiovascular risk, patients with CVD were more likely to be overweight, had a lower diastolic blood pressure, had higher serum triglycerides, and lower serum HDL-cholesterol. No differences were observed in systolic blood pressure, total and LDL cholesterol levels, smoking status, and use of alcohol. Furthermore, subjects with CVD were more likely to have a chronic lung disease, diabetes or urine incontinence, used a larger number of drugs and were more likely to take antihypertensive agents and lipid lowering drugs.

**Table 2:** Cross-sectional association of CVD, other co-morbidities, polypharmacy with frailty risk (n=1432).

	Odds ratio	95% CI	p-value
CVD^a^			
Combined^b^	2.11	1.39-3.21	0.00
AP	2.09	1.23-3.55	0.01
MI	1.92	0.48-7.59	0.36
HF	2.66	1.44-4.92	0.00
Stroke	1.60	0.47-5.45	0.45
PAD	3.50	1.01-12.12	0.05

CVD^c^			
Combined^b^	1.77	1.13-2.75	0.01
AP	1.65	0.94-2.89	0.08
MI	2.43	0.57-10.39	0.23
HF	2.13	1.15-4.08	0.02
Stroke	1.79	0.51-6.32	0.37
PAD	4.08	1.07-15.61	0.04

Chronic diseases			
Lung disease	1.85	1.26-2.72	0.00
Arthritis	2.22	1.59-3.09	0.00
Cancer	1.12	0.70-1.30	0.65
Diabetes	1.49	0.91-2.44	0.11
Urine incontinence	2.48	1.79-3.44	0.00

Polypharmacy^d^	3.75	2.64-5.32	0.00

CVD=Cardiovascular disease, HF=Heart failure, AP=Angina pectoris, MI=Myocardial infarction, PAD=Peripheral artery disease.

a Adjusted for age and sex.

b AP, MI, HF, stroke, PAD combined.

c Adjusted for age, sex, lung disease, arthritis, urine incontinence.

d Taking 5 or more drugs.

Of the 1432 subjects included in the analyses 210 (15%) were frail at baseline. Of these, 42 (20%) had CVD. In the age and sex adjusted models, subjects with CVD were all more likely to be frail than subjects without CVD (table 2). Odds ratios (ORs) varied from 1.92 to 3.50 and reached statistically significance in the analyses with any CVD, AP, HF, and PAD. These effect estimates were all comparable to the estimates of the relation between e.g. polypharmacy or arthritis with frailty with ORs (95%CI) of 3.75 (2.64-5.32), and 2.22 (1.59-3.09). In secondary analyses (table 2), we also adjusted our model for the comorbidities which were strongest associated with frailty (chronic lung disease, arthritis, and incontinence). The results of these analyses did not differ significantly from the age and sex adjusted model. After adding polypharmacy to the comorbidities adjusted model (results not presented in table), the effect between CVD and frailty disappeared (HR (95% CI) 1.10 (0.68-1.76)). This is probably due to the high correlation between frailty, CVD and polypharmacy and explains why polypharmacy is also often used as a surrogate marker for frailty. Because adding polypharmacy to the models leads to over adjustment, we only adjusted our secondary cross-sectional and longitudinal analyses for age, sex, and comorbidities.

**Table 3:** Longitudinal association between CVD and frailty, excluding subject’s frail at baseline (n=1222).

	Hazard ratio	95% CI	p-value
CVD^a^			
Combined^b^	1.41	0.95-2.08	0.09
AP	1.21	0.72-2.05	0.47
MI	0.57	0.14-2.29	0.43
HF	2.28	1.27-4.08	0.01
Stroke	1.76	0.56-5.01	0.33
PAD	1.59	0.39-6.40	0.52

CVD^c^			
Combined^b^	1.36	0.92-2.00	0.12
AP	1.18	0.70-1.99	0.54
MI	0.58	0.14-2.32	0.44
HF	2.09	1.14-3.83	0.02
Stroke	1.87	0.59-5.56	0.28
PAD	1.81	0.45-7.24	0.40

Chronic diseases			
Lung disease	1.40	1.01-1.93	0.04
Arthritis	1.70	1.33-2.16	0.00
Cancer	1.01	0.68-1.48	0.97
Diabetes	1.75	1.14-2.68	0.01
Urine incontinence	1.14	0.87-1.51	0.34

Polypharmacy^d^	1.88	1.36-2.59	0.00

CVD=Cardiovascular disease, HF=Heart failure, AP=Angina pectoris, MI=Myocardial infarction, PAD=Peripheral artery disease.

a Adjusted for age and sex.

b AP, MI, HF, stroke, PAD combined.

c Adjusted for age, sex, lung disease, arthritis, urine incontinence.

d Taking 5 or more drugs.

### Longitudinal association between CVD and incident frailty

Non-frail subjects with and without CVD were included in the longitudinal analyses between CVD and incident frailty (n=1222). Subjects with CVD (n=106) were older, more often male, had higher triglyceride levels, lower HDL-cholesterol levels, and were more often former smokers than non-frail subjects without CVD (n=1116) (supplemental table 1). Further, no differences in nutritional status, chronic lung disease, arthritis, cancer, diabetes or urine incontinence were seen, but patients with CVD were more likely to take at least two drugs.

Subjects with CVD had a shorter median follow-up (8.4 years; range: 4.7-8.4) than subjects without CVD (12.2 years; range: 6.2-18.6) (p-value 0.00). During follow-up 285 (23%) subjects became frail. Hazard ratios (HRs) and 95%CI for incident frailty, adjusted for age and sex, are presented in table 3. Although not all statistically significant, subjects with any CVD, AP, stroke, and especially those with HF and PAD were more likely to become frail than patients without CVD, respectively AP, stroke, HF and PAD during follow-up (HRs ranging from 1.21 to 2.28). Only the associations in patients with HF were statistically significant; they were at least twice as likely to become frail than their peers without HF (HR 2.28; 95%CI: 1.27-4.08). These effect estimates were comparable to the estimates of the relation between chronic lung disease, arthritis, diabetes or polypharmacy with incident frailty risk; HRs (95%CI) were 1.40 (1.01-1.93), 1.70 (1.33-2.16), 1.75 (1.14-2.68), and 1.88 (1.36-2.59). The number of non-frail patients with each specific CVD that became frail during follow-up are presented in supplemental table 3. In secondary analyses (table 3), we added chronic lung disease, arthritis, and incontinence to the age and sex adjusted model. These results were similar to the results of the age and sex adjusted model; only the associations in patients with HF were statistically significant. As described in the paragraph listing the results of the cross-sectional analyses, polypharmacy is too highly correlated with frailty and can be seen as a mediating variable in the association between CVD and frailty. Thus, we did not add polypharmacy to the secondary model.

### Longitudinal association between frailty and incident CVD

Subjects without CVD at baseline were included in the longitudinal analyses between frailty and incident CVD risk (n=1284). Subjects who were frail at baseline (n=168) were older, more often female, had a lower diastolic blood pressure, lower serum levels of total cholesterol and LDL cholesterol, smoked less, and drank less alcohol then those not frail (n=1116) (supplemental table 2). They were more likely to have a low weight, have chronic lung disease, arthritis, urine incontinence, and to take 2 or more drugs. No baseline differences were observed in systolic blood pressure, HDL cholesterol, triglyceride levels, cancer, and diabetes. At the first follow-up cycle, 131 subjects without CVD at baseline had been diagnosed with CVD during the three years preceding this follow-up. Cox regression analysis, adjusted for age and sex, showed that frailty was not statistically significantly related to incident CVD risk (HR 1.47; 95%CI: 0.86-2.52) (results not reported in table).

### Time-lag analyses between CVD and incident frailty

In the longitudinal analyses, excluding those developing frailty within the first 5 years (n=1133), CVD was associated with an increased risk of frailty (HR 1.08; 95%CI: 0.65-1.79). Focusing on the individual CVDs, of the 27 HF patients with available data, 4 (15%) became frail at a later point in time. They got frail more often than participants without HF (HR 11.0; 95%CI: 3.79-31.78).

Because no association between frailty and incident CVD was observed, no time-lag analysis was performed to study if frailty preceded CVD in the longer term.

## DISCUSSION

The present study studied the bidirectional effect of CVD on frailty among community-dwelling older adults. First, we observed cross-sectional associations between CVD and frailty. Patients with CVD, especially those with PAD and HF, were more likely to be frail. Longitudinally, mainly HF was associated with incident frailty. These patients were at least twice as likely to become frail, which puts these patients at an equal or even higher risk of incident frailty than subjects with chronic lung disease, arthritis or diabetes. Analyses studying the reverse association revealed that in this older population, frailty does not precede development of CVD during three years of follow-up.

Our findings are in line with previous studies on the relation between CVD and frailty showing that CVD was associated with frailty in community-dwelling men and women in cross-sectional data [10, 32, 33]. Longitudinal evidence is however not as abundant. In the Women’s Health Initiative Observational Study (WHI-OS) [25] coronary artery disease, diabetes, and hypertension were all specifically associated with incident frailty after three years of follow-up. Our study adds to these results because we studied a broader range of specific CVDs and had a longer follow-up.

In this study, results of the analyses with any CVD and PAD and incident frailty were suggestive of existing associations, although not statistically significant. Nonetheless, the magnitude of the positive HRs, ranging from 1.21 in patients with AP to 2.28 in patients with HF, make the association between CVD and incident frailty likely. In terms of causality, the lack of statistically significant associations and small HRs in the longitudinal associations between frailty and incident CVD suggest CVD precedes frailty, rather than frailty preceding CVD. Specifically, the time-lag analysis shows that particularly HF patients are at increased risk frailty. However, this must be interpreted with caution considering the small number of HF patients.

Existing literature recognizes that patients with HF are more often frail [34, 35]. However, much less is known about the effect of HF on frailty over time. Our study with a follow-up of 17 years illustrates that patients with HF, of all patients with CVDs, are at greatest risk of frailty. Treatment regimes in this vulnerable group of patients should call for a shift from ‘problem-based, disease-oriented’ care aiming at improvement of outcomes to ‘goal-oriented, integrated’ care in which quality of life and independency of (health) care become of increasing importance [36].

A number of strengths and weaknesses of this study should be acknowledged. Key strengths include the design with a long follow-up for frailty (17 years). Furthermore, the assessment of frailty is based on established instruments and objective measurements, in contrast to many other frailty measurements, which are only based on questionnaires. This study is limited by the three years between follow-up cycles. Hence, it is not possible to ascertain at what exact moment in time a subject has become frail. However, this is inherent in most prospective observational studies and an inevitable consequence of the research design. Also, this study was limited in the length of follow-up for incident CVD (three years) and the relatively low number of patients categorized as having CVD. The latter is probably due to the CVD algorithm used in this study which probably underestimated the actual prevalence of CVD in these older adults. The short follow-up for incident CVD may explain the lack of a longitudinal association between frailty and incident CVD.

This study set out to examine the bidirectional longitudinal association between CVD and frailty risk over an extended period of time. In conclusion, older adults with HF have an increased risk of frailty and frail older adults do not have an increased risk of CVD. These findings emphasize that treatment regimes in these vulnerable patients should mainly focus on improving quality of life and independence of care instead of focusing on curative aspects of treatment. It also highlights the need for prospective cardiac rehabilitation programs evaluating the effect of physical exercise programs in order to prevent frailty. With this, quality of life and independence of care could be improved, which are important aspects of care in such vulnerable patients.

## Supplemetary Material

The Supplemenatry material for this article can be found online at:
www.aginganddisease.org/EN/10.14336/AD.2017.1125
**Supplemental table 1**. Baseline characteristics in subjects with and without CVD, excluding subject’s frail at baseline (n=1222).
**Supplemental table 2**. Baseline characteristics in frail and non-frail subjects, excluding subjects with CVD (n=1284).
**Supplemental table 3**. Number of non-frail CVD patients that became frail during follow-up.
