# Supplementary material for: Frailty in Older Adults with Cardiovascular Disease: Cause, Effect or Both?
Source: Aging Dis. 2018 Jun 1;9(3):489–97. doi: 10.14336/AD.2017.1125 (PMC5988603; doi:10.14336/AD.2017.1125)
Supplement: Supplementary file 1 — The Supplemenatry material for this article can be found online at: www.aginganddisease.org/EN/10.14336/AD.2017.1125 Supplemental table 1. Baseline characteristics in subjects with and without CVD, excluding subject’s frail at baseline (n=1222). Supplemental table 2. Baseline characteristics in frail and non-frail subjects, excluding subjects with CVD (n=1284). Supplemental table 3. Number of non-frail CVD patients that became frail during follow-up. [file ad-9-3-489-s001.zip › AD-9-3-489-s001/ad-9-3-489-s001.docx]

**Frailty in Older Adults with Cardiovascular Disease: Cause, Effect or Both?**

**Emma EF. Kleipool^1^, Emiel O. Hoogendijk^2^, Marijke C. Trappenburg^1^, M. Louis Handoko^3^, Martijn Huisman^2,4^, Mike JL. Peters^1^, Majon Muller^1,*^**

^1^Department of Internal medicine and Geriatrics, VU University Medical Center, 1081 HV Amsterdam, The Netherlands

^2^Department of Epidemiology and Biostatistics, Amsterdam Public Health research institute, VU University Medical Center, 1007 MB Amsterdam, The Netherlands

^3^Department of Cardiology, VU University Medical Center, 1081 HV Amsterdam, The Netherlands

^4^Department of Sociology, VU University, 1081 HV Amsterdam, The Netherlands

| **Supplemental table 1.** Baseline characteristics in subjects with and without CVD, excluding subject’s frail at baseline (n=1222). |
| --- |
| \|  \| **CVD** \| \| **P-value** \| \| --- \| --- \| --- \| --- \| \| **Yes**  N=106 \| **No**  N=1116 \| \| **Demographics** \|  \|  \|  \| \| Age (yrs)^a^ \| 77.6 ± 6.1 \| 74.6 ± 6.4 \| 0.00 \| \| Sex (% female) \| 37% \| 51% \| 0.01 \| \| **Cardiovascular disease**^b^ \|  \|  \|  \| \| Angina pectoris \| 59 (56%) \|  \|  \| \| Myocardial infarction \| 13 (12%) \|  \|  \| \| Heart failure \| 34 (32%) \|  \|  \| \| Stroke \| 10 (9%) \|  \|  \| \| Peripheral artery disease \| 9 (9%) \|  \|  \| \| **Cardiovascular risk factors** \|  \|  \|  \| \| Nutritional status^b^ \|  \|  \| 0.08 \| \| Low weight (BMI^c^ <20) \| 1 (1%) \| 40 (4%) \|  \| \| Normal weight (BMI 20-25) \| 27(25%) \| 349 (31%) \|  \| \| Overweight (BMI >25) \| 78 (74%) \| 727 (65%) \|  \| \| Systolic blood pressure (mmHg)^a^ \| 151 ± 29 \| 154 ± 26 \| 0.53 \| \| Diastolic blood pressure (mmHg)^a^ \| 82 ± 18 \| 84 ± 13 \| 0.33 \| \| Serum cholesterol (mmol/L)^a^ \|  \|  \|  \| \| Total cholesterol \| 5.6 ± 0.9 \| 5.7 ± 1.0 \| 0.26 \| \| LDL cholesterol \| 3.6 ± 0.9 \| 3.7 ± 0.9 \| 0.45 \| \| HDL cholesterol \| 1.2 ± 0.4 \| 1.4 ± 0.4 \| 0.01 \| \| Triglycerides \| 1.6 ± 0.8 \| 1.4 ± 0.8 \| 0.04 \| \| Smoking^b^  Never  Former  Current \| 27 (26%)  65 (62%)  13 (12%) \| 378 (34%)  519 (46%)  218 (20%) \| 0.01 \| \| Alcohol use^b^  No  Light  Moderate/excessive \| 27 (26%)  54 (51%)  24 (23%) \| 238 (21%)  567 (51%)  310 (28%) \| 0.43 \| \| **Chronic diseases**^b^ \|  \|  \|  \| \| Chronic lung disease \| 21 (20%) \| 155 (14%) \| 0.10 \| \| Arthritis \| 46 (43%) \| 493 (44%) \| 0.88 \| \| Cancer \| 10 (9%) \| 138 (12%) \| 0.38 \| \| Diabetes mellitus \| 11 (10%) \| 78 (7%) \| 0.20 \| \| Urine incontinence \| 32 (30%) \| 249 (22%) \| 0.07 \| \| **Medication**  No. of drugs taken  0  1  ≥2  Antihypertensive drugs^b^  Lipid lowering drugs^b^ \| 3 (3%)  4 (4%)  99 (93%)  88 (83%)  12 (11%) \| 328 (29%)  261 (24%)  527 (47%)  373 (33%)  47 (4%) \| 0.00  0.00  0.00 \| |
| ^a^ Mean ± standard deviation is presented.  ^b^ Number of subjects (%).  ^c^ Body mass index (kg/m^2^). |

| **Supplemental table 2.** Baseline characteristics in frail and non‑frail subjects, excluding subjects with CVD (n=1284). |
| --- |
| \|  \| **Frail** \| \| **P-value** \| \| \| --- \| --- \| --- \| --- \| --- \| \| **Yes**  N=168 \| **No**  N=1116 \| \| **Demographics** \|  \|  \| \|  \| \| Age (yrs)^a^ \| 80.4 ± 6.0 \| 74.6 ± 6.4 \| \| 0.00 \| \| Sex (% female) \| 60% \| 40% \| \| 0.04 \| \| **Cardiovascular risk factors** \|  \|  \| \|  \| \| Nutritional status^b^ \|  \|  \| \| 0.04 \| \| Low weight (BMI^c^ <20) \| 13 (8%) \| 42 (4%) \| \|  \| \| Normal weight (BMI 20-25) \| 49 (27%) \| 353 (31%) \| \|  \| \| Overweight (BMI >25) \| 106 (65%) \| 729 (65%) \| \|  \| \| Systolic blood pressure (mmHg)^a^ \| 152 ± 27 \| 154 ± 26 \| \| 0.26 \| \| Diastolic blood pressure (mmHg)^a^ \| 81 ± 14 \| 84 ± 13 \| \| 0.01 \| \| Serum cholesterol (mmol/L)^a^ \|  \|  \| \|  \| \| Total cholesterol \| 5.5 ± 1.2 \| 5.7 ± 1.0 \| \| 0.02 \| \| LDL cholesterol \| 3.5 ± 1.1 \| 3.7 ± 0.9 \| \| 0.02 \| \| HDL cholesterol \| 1.3 ± 0.4 \| 1.4 ± 0.4 \| \| 0.31 \| \| Triglycerides \| 1.5 ± 0.8 \| 1.4 ± 0.7 \| \| 0.96 \| \| Smoking^b^  Never  Former  Current \| 81 (48%)  51 (30%)  36 (22%) \| 378 (34%)  519 (46%)  218 (20%) \| \| 0.01 \| \| Alcohol use^b^  No  Light  Moderate/excessive \| 73 (44%)  74 (44%)  21 (12%) \| 238 (21%)  567 (51%)  310 (28%) \| \| 0.00 \| \| **Chronic diseases**^b^ \|  \|  \| \|  \| \| Chronic lung disease \| 33 (20%) \| 155 (14%) \| \| 0.05 \| \| Arthritis \| 109 (65%) \| 493 (44%) \| \| 0.00 \| \| Cancer \| 18 (11%) \| 138 (12%) \| \| 0.54 \| \| Diabetes mellitus \| 17 (18%) \| 78 (7%) \| \| 0.15 \| \| Urine incontinence \| 83 (49%) \| 249 (22%) \| \| 0.00 \| \| **Medication**^b^  No. of drugs taken  0  1  ≥2  Antihypertensive drugs  Lipid lowering drugs \| 23 (14%)  26 (16%)  119 (71%)  83 (49%)  4 (2%) \| 328 (29%)  261 (23%)  527 (47%)  373 (33%)  47 (4%) \| \| 0.00  0.00  0.26 \| |
| ^a^ Mean ± standard deviation is presented.  ^b^ Number of subjects (%).  ^c^ Body mass index (kg/m^2^). |

| **Supplemental table 3.** Number of non-frail CVD patients that became frail during follow-up. |
| --- |
| \| **Specific CVD** \| **Number at baseline** \| **Incident frail (%)** \| \| --- \| --- \| --- \| \| Angina pectoris \| 59 \| 15 (25%) \| \| Myocardial infarction \| 13 \| 2 (15%) \| \| Heart failure \| 34 \| 11 (32%) \| \| Stroke \| 10 \| 3 (30%) \| \| Peripheral artery disease \| 9 \| 3 (33%) \| |
|  |
