# Supplementary material for: Frailty in Older Adults with Cardiovascular Disease: Cause, Effect or Both?
Source: Aging Dis. 2018 Jun 1;9(3):489–97. doi: 10.14336/AD.2017.1125 (PMC5988603; doi:10.14336/AD.2017.1125)
Supplement: Supplementary file 1 — The Supplemenatry material for this article can be found online at: www.aginganddisease.org/EN/10.14336/AD.2017.1125 Supplemental table 1. Baseline characteristics in subjects with and without CVD, excluding subject’s frail at baseline (n=1222). Supplemental table 2. Baseline characteristics in frail and non-frail subjects, excluding subjects with CVD (n=1284). Supplemental table 3. Number of non-frail CVD patients that became frail during follow-up. [file ad-9-3-489-s001.zip › AD-9-3-489-s001/ad-9-3-489-s001.pdf]

SUPPLEMENTARY DATA

Frailty in Older Adults with Cardiovascular Disease: Cause, Effect or Both?

Emma EF. Kleipool[1], Emiel O. Hoogendijk[2], Marijke C. Trappenburg[1], M. Louis Handoko[3], Martijn Huisman[2,4], Mike JL. Peters[1], Majon Muller[1,*]

[1]Department of Internal medicine and Geriatrics, VU University Medical Center, 1081 HV Amsterdam, The Netherlands
[2]Department of Epidemiology and Biostatistics, Amsterdam Public Health research institute, VU University Medical Center, 1007 MB Amsterdam, The Netherlands
[3]Department of Cardiology, VU University Medical Center, 1081 HV Amsterdam, The Netherlands
[4]Department of Sociology, VU University, 1081 HV Amsterdam, The Netherlands

© 2017 Kleipool EEF et al. Published online at www.aginganddisease.org/EN/10.14336/AD.2017.1125

Supplemental table 1. Baseline characteristics in subjects with and without CVD, excluding subject's frail at baseline (n=1222).

	CVD		P-value
	**Yes** N=106	**No** N=1116	
**Demographics**			
Age (yrs)[a]	77.6 ± 6.1	74.6 ± 6.4	0.00
Sex (% female)	37%	51%	0.01
**Cardiovascular disease**[b]			
Angina pectoris	59 (56%)		
Myocardial infarction	13 (12%)		
Heart failure	34 (32%)		
Stroke	10 (9%)		
Peripheral artery disease	9 (9%)		
**Cardiovascular risk factors**			
Nutritional status[b]			0.08
Low weight (BMI[c] <20)	1 (1%)	40 (4%)	
Normal weight (BMI 20-25)	27(25%)	349 (31%)	
Overweight (BMI >25)	78 (74%)	727 (65%)	
Systolic blood pressure (mmHg)[a]	151 ± 29	154 ± 26	0.53
Diastolic blood pressure (mmHg)[a]	82 ± 18	84 ± 13	0.33
Serum cholesterol (mmol/L)[a]			
Total cholesterol	5.6 ± 0.9	5.7 ± 1.0	0.26
LDL cholesterol	3.6 ± 0.9	3.7 ± 0.9	0.45
HDL cholesterol	1.2 ± 0.4	1.4 ± 0.4	0.01
Triglycerides	1.6 ± 0.8	1.4 ± 0.8	0.04
Smoking[b]			0.01
Never	27 (26%)	378 (34%)	
Former	65 (62%)	519 (46%)	
Current	13 (12%)	218 (20%)	
Alcohol use[b]			0.43
No	27 (26%)	238 (21%)	
Light	54 (51%)	567 (51%)	
Moderate/excessive	24 (23%)	310 (28%)	
**Chronic diseases**[b]			
Chronic lung disease	21 (20%)	155 (14%)	0.10
Arthritis	46 (43%)	493 (44%)	0.88
Cancer	10 (9%)	138 (12%)	0.38
Diabetes mellitus	11 (10%)	78 (7%)	0.20
Urine incontinence	32 (30%)	249 (22%)	0.07
**Medication**			
No. of drugs taken			0.00
0	3 (3%)	328 (29%)	
1	4 (4%)	261 (24%)	
≥2	99 (93%)	527 (47%)	
Antihypertensive drugs[b]	88 (83%)	373 (33%)	0.00
Lipid lowering drugs[b]	12 (11%)	47 (4%)	0.00

[a] Mean ± standard deviation is presented.
[b] Number of subjects (%).
[c] Body mass index (kg/m^2).

SUPPLEMENTARY DATA

Supplemental table 2. Baseline characteristics in frail and non-frail subjects, excluding subjects with CVD (n=1284).

	Frail		P-value
	**Yes** N=168	**No** N=1116	
**Demographics**			
Age (yrs)[a]	80.4 ± 6.0	74.6 ± 6.4	0.00
Sex (% female)	60%	40%	0.04
**Cardiovascular risk factors**			
Nutritional status[b]			0.04
Low weight (BMI[c] <20)	13 (8%)	42 (4%)	
Normal weight (BMI 20-25)	49 (27%)	353 (31%)	
Overweight (BMI >25)	106 (65%)	729 (65%)	
Systolic blood pressure (mmHg)[a]	152 ± 27	154 ± 26	0.26
Diastolic blood pressure (mmHg)[a]	81 ± 14	84 ± 13	0.01
Serum cholesterol (mmol/L)[a]			
Total cholesterol	5.5 ± 1.2	5.7 ± 1.0	0.02
LDL cholesterol	3.5 ± 1.1	3.7 ± 0.9	0.02
HDL cholesterol	1.3 ± 0.4	1.4 ± 0.4	0.31
Triglycerides	1.5 ± 0.8	1.4 ± 0.7	0.96
Smoking[b]			0.01
Never	81 (48%)	378 (34%)	
Former	51 (30%)	519 (46%)	
Current	36 (22%)	218 (20%)	
Alcohol use[b]			0.00
No	73 (44%)	238 (21%)	
Light	74 (44%)	567 (51%)	
Moderate/excessive	21 (12%)	310 (28%)	
**Chronic diseases**[b]			
Chronic lung disease	33 (20%)	155 (14%)	0.05
Arthritis	109 (65%)	493 (44%)	0.00
Cancer	18 (11%)	138 (12%)	0.54
Diabetes mellitus	17 (18%)	78 (7%)	0.15
Urine incontinence	83 (49%)	249 (22%)	0.00
**Medication**[b]			
No. of drugs taken			0.00
0	23 (14%)	328 (29%)	
1	26 (16%)	261 (23%)	
≥2	119 (71%)	527 (47%)	
Antihypertensive drugs	83 (49%)	373 (33%)	0.00
Lipid lowering drugs	4 (2%)	47 (4%)	0.26

[a] Mean ± standard deviation is presented.
[b] Number of subjects (%).
[c] Body mass index (kg/m^2).

© 2017 Kleipool EEF et al. Published online at www.aginganddisease.org/EN/10.14336/AD.2017.1125

SUPPLEMENTARY DATA

Supplemental table 3. Number of non-frail CVD patients that became frail during follow-up.

Specific CVD	Number at baseline	Incident frail (%)
Angina pectoris	59	15 (25%)
Myocardial infarction	13	2 (15%)
Heart failure	34	11 (32%)
Stroke	10	3 (30%)
Peripheral artery disease	9	3 (33%)

© 2017 Kleipool EEF et al. Published online at www.aginganddisease.org/EN/10.14336/AD.2017.1125
